# Microplastic Pollution: A Global Environmental Crisis Impacting Marine Life, Human Health, and Potential Innovative Sustainable Solutions

**DOI:** 10.3390/ijerph22060889

**Published:** 2025-06-02

**Authors:** Prithviraj Karak, Afsona Parveen, Anindya Modak, Atin Adhikari, Sankha Chakrabortty

**Affiliations:** 1 Department of Physiology, Bankura Christian College, Bankura 722101, India; 2 Department of Bachelor in Medical Laboratory Technology, Durgapur Institute of Paramedical Science, Durgapur 713212, India; afsana.parveen321@gmail.com; 3 Department of Human Physiology, University of Calcutta, Kolkata 700009, India; anindyamodak78@gmail.com; 4 Department of Biostatistics, Epidemiology, and Environmental Health Sciences, Jiann-Ping Hsu College of Public Health, Georgia Southern University, Statesboro, GA 30460, USA; 5 School of Chemical Engineering, KIIT Deemed to be University, Bhubaneswar 751024, India; sankha.nit@gmail.com

**Keywords:** microplastic, harmful effects, marine organisms, cellular damage, oxidative stress, treatment technologies

## Abstract

Pollution, especially plastic pollution, presents a serious worldwide danger to essential environmental resources. Microplastics are tiny plastic fragments varying in size from 50 μm to 5 mm. The primary aim of this article is to develop an extensive review grounded in the latest data accessible until 2024, adhering to PRISMA guidelines. A total of 329 data points were collected and 297 of those were removed through filtering, leaving 32 articles for the study, and taking into account the complete evolution of all the publications. This study seeks to enhance public awareness and knowledge among researchers about the harmful effects of plastic pollution on the environment and society by identifying its sources and consequences for humans and ecosystems. A detailed analysis of the sources of microplastics in the oceans and their detrimental effects on marine organisms is presented. This research additionally explores the transport of microplastics through various environmental pathways, including water and air. Aquatic species ingest microplastics, which subsequently transfer up the food chain, including humans, and these risks are discussed. Microplastics may increase the production of reactive oxygen species (ROS), leading to DNA and cellular damage, oxidative stress, alterations in gene expression, and decreased cell viability. Developing clear and effective guidelines and regulations is crucial for addressing the adverse issues related to microplastics. All participants in the policymaking and implementation of these guidelines must understand their roles and responsibilities.

## 1. Introduction

Synthetic polymers with pliable or malleable (flexible) properties that allow for shape-molding are known as plastics. Long polymer chains made of carbon, oxygen, hydrogen, silicon, and chloride—all of which come from coal, oil, and natural gas—make up plastic. Pollution, particularly plastic pollution, poses a grave global threat to vital environmental resources. Currently, the whole world is facing several kinds of pollution, among which plastic pollution is also a notable one [1]. Unfortunately, the widespread usage of plastic and its byproducts results in a significant amount of waste plastics, which are released into the environment untreated [1]. The definition of plastics is “polymeric material that may contain other substances to improve performances and/or reduce costs”. As a material, polymeric material has existed over the past century [2], and the mass production of plastic began in 1950s [3].

The number of plastic items produced annually worldwide was around 460 million tons in 2019, with 9% going toward recycling. By 2060, that amount is predicted to rise to 1.2 billion tons [4]. Every year, almost 8 million tons of plastic waste from the land is discarded into the ocean [4], of which 1% is made up entirely of minute plastic trash. Because of their convenience and remarkable cost-to-performance ratios, plastics, a material that has a number of uses, have proven crucial in preserving the comfort and quality of contemporary living [5]. They are composed of a variety of high molecular weight organic chemicals, including ethylene, vinyl chloride, vinyl acetate, vinyl alcohol, and so on. Because plastic is flexible, it may be molded into a variety of forms when it is soft and then solidify into a hard or somewhat flexible item or a solid object of any size or shape [6].

Plastics are synthetic polymers, persistent in the environment, and do not break readily. Significant environmental contamination issues have been brought on by their discharge into the environment [7]. The amount of plastic garbage in the environment is a major concern since it may have a negative impact on humans, wildlife, and their environments. One of the main environmental issues at the moment is plastic garbage, which has been abundantly demonstrated by several studies in recent decades to exist and have an influence on a variety of natural regions [8,9]. Plastic garbage is still piling up at various trophic levels of the ecosystem, despite increased awareness of the problem on a global scale. Plastics are mostly used and produced in large quantities. After usage, plastic and its byproducts are often discarded into the environment [10]. According to previous studies, the amount of plastic garbage created in 2010 by 192 coastal nations was around 275 million tons, surpassing the total amount of plastic and its products produced worldwide [11]. India produces 9.3 million tons of coal annually, followed by Nigeria (3.5 million tons), Indonesia (3.4 million tons), China (2.8 million tons), Pakistan (2.6 million tons), Bangladesh (1.7 million tons), Russia (1.7 million tons), Brazil (1.4 million tons), Thailand (1.0 million tons), and the Democratic Republic of the Congo (1.0 million tons) [12]. As a whole, these countries produce a substantial amount of coal each year. Furthermore, an alternative study team discovered that around 12.7 million tons of plastic debris enters the seas and oceans annually [13].

### 1.1. Types of Microplastics, Uses, and Sources

Based on their particle size and the kinds of materials and components used in their production, plastics may be broadly classified into two groups, which are listed in Table 1. “Macroplastics” are plastic materials that are typically larger than 5 mm in their largest diameter. “Microplastics” are plastic items and garbage that are less than 5 mm in size and have the ability to break into numerous smaller pieces [14,15]. In the middle of the 2000s, the idea of “microplastics” first appeared, considering plastic particles with sizes between 50 μm and 5 mm [16]. The British marine researcher Professor Richard Thompson of the University of Plymouth first used the term “microplastic” in 2004. Later, a relatively new area of environmental research focused on the many impacts of nanoplastics, which are plastic particles that are smaller than 100 nm [17,18]. Plastics can persist in the environment as both microplastics and nanoplastics. They are identified as emergent particle anthropogenic pollutants and may be found in the environment, including soil, drinking water, ground water, ocean, sediments, air, and in different biota.

Prior research on microplastic pollution was limited in scope, and there was a lack of standardized procedures, data on the sources of microplastics, and analytical methods. In order to overcome these shortcomings, this review performed a thorough search of the literature, examined the distribution of sources, suggested a standard procedure for sampling, extraction, and analysis, and investigated cutting-edge analytical methods such as mass spectrometry and Raman spectroscopy. Utilizing a systematic review approach, quality evaluation, and data synthesis, the review checks for bias, integrates the results of the included studies, and ensures a thorough and transparent literature search.

### 1.2. Objective of This Study

The main objectives of this review are to identify the sources, causes, and effects of microplastics, evaluate their prevalence in both humans and the environment, and explore our potential contributions to treatment and mitigation strategies. Alongside this comprehensive examination of plastic’s impact on people and the planet, our campaigns in this article seek to raise societal awareness about the detrimental effects of microplastics.

This review article offers a distinctive perspective on microplastic pollution by synthesizing insights from marine biology, environmental science, and public health, reflecting the latest research available. It highlights innovative solutions and cutting-edge technologies aimed at addressing the challenge of microplastic pollution. Additionally, the review provides a global overview, drawing attention to regional disparities and potential interventions. By exploring the interconnections between microplastic pollution, marine ecosystems, human health, and sustainable solutions, it creates a comprehensive synthesis of current knowledge and future pathways. This holistic viewpoint may enable academics, policymakers, and stakeholders to gain a deeper understanding of the complexities surrounding microplastic pollution and to formulate more effective strategies for its mitigation.

## 2. Materials and Methods

The meta-analyses criteria, systematic reviews, and checklists were followed in the planning and implementation of this systematic review.

### 2.1. Search Strategy

For the systematic reviews, a variety of search engines were employed, including PubMed, MEDLINE, and NDSL (National Digital Science Library). Search engines were used to gather data by searching for specific phrases like “microplastic and environment, microplastic pollution”, “microplastic toxicity and health hazards”, “properties and types of microplastic”, and “molecular mechanism of microplastic in human body”. Articles between 2000 and 2024 were included in this study. The Preferred Reporting Items for Systematic Reviews and Meta-Analysis (PRISMA) guidelines were followed throughout the literature search and review procedure.

### 2.2. Study Inclusion and Exclusion Criteria

Articles written in English and published in peer-reviewed journals that met the specified criteria for population size, amount of exposure in the human body, result outcome after exposure, and study design of our relevant topic were included in this review. Research articles that indicated the potential for human exposure through the food chain and that demonstrated the potential for health impacts by extrapolating the results of animal trials to the human body were included in the review. Studies that reported on endpoints such as mortality, growth inhibition, or biochemical changes in organisms exposed to microplastics were included. Physical characterization methods include microscopy and particle size analysis, while chemical characterization methods include spectroscopy and chromatography.

Exclusions include studies that lack sufficient detail or use widely accepted methods. Exclusions include studies using validated methods or lacking sufficient detail on method parameters and quality control. Studies that did not provide sufficient information on microplastic characterization or toxic endpoints, studies including review articles or conference abstracts without original data, studies that were not written in English, and studies that did not meet the predefined quality thresholds were excluded from this review.

## 3. Results

### Selection of Review Articles

**Figure 1:**
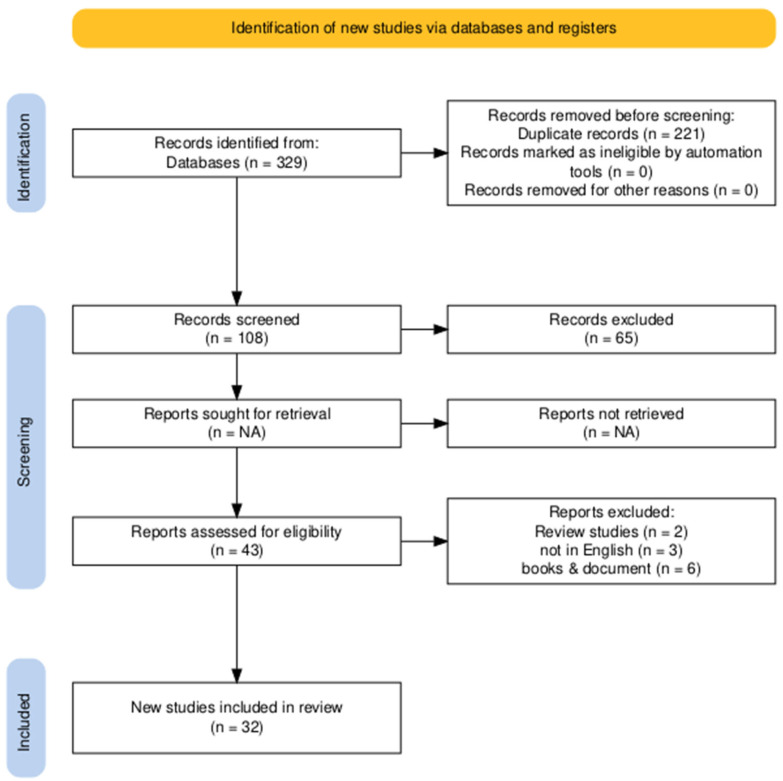
PRISMA flowchart for study selection and screening.

In this review, we tried to enhance the methodology for identifying and choosing research in accordance with the PRISMA 2020 guidelines (Figure 1). Approximately 329 data points were gathered from all reliable search engines; 221 duplicate data points were eliminated by filtering, resulting in the discovery of 108 databases. Of those, forty-three data points were determined to be suitable for this investigation after sixty-five data points had been eliminated based on exclusion criteria. Thirty-two articles remained for the study, considering the whole evolution of all the publications, with eleven original articles removed in Table 2 [19,20,21,22,23,24,25,26,27,28,29,30,31,32,33,34,35,36,37,38,39,40,41,42,43,44,45,46,47,48,49,50]. Table 3 provides additional features of the chosen research articles along with their purposes and findings.

## 4. Discussion

### 4.1. Environmental Microplastic Pollution

Microplastic pollution has been identified in various environmental areas. Very small plastic particles, about one millimeter in size, have been found floating on the surface of seawater, with a concentration of approximately 1.1 g per square kilometer [51]. In sea-sides and coastlines, where microplastics usually assemble and are abundant, levels have been found to vary from 27 to 5595 particles per square meter [52,53,54]. Additionally, recent research discovered significantly high concentrations of microplastics (0.58–2116 pieces/kg) in low-energy sludge layers [55]. The concentration of microplastics near the continental shelf’s sea surface ranges from 3 to 102,000 particles per cubic meter (m^3^) [56,57]. Microplastics, ranging from 0 to 400 particles/m^3^, have been found in sediments taken at depths of 1176–4844 m in the deep sea. Because microplastics are so common in the deep sea, pollution can have an impact on a variety of marine life types. Different trophic levels consume these affected aquatic species, and eventually, higher trophic levels like humans are affected as well. Several investigations reveal that 83% of the lobsters consumed microplastics [57]. According to Cheung et al., marine organisms in China consume relatively little microplastic and its products [58]. Another study shows that microplastics were present in the tissues of all shellfish sampled from the Chinese market, with 4–57 particles detected per sample [59]. Another research group found that microplastic was also found in wild oysters in the Pearl River Estuary, with 1–7 particles detected per oyster [60]. Plastic pellets used by manufacturers, tires from automobiles, and synthetic apparel are sources of microplastics. They are also produced by the physical decomposition of plastic garbage. Rainwater carries them into lakes, ponds, rivers, and the ocean; in addition, treated sewage waste that is applied as fertilizer to fields can also carry them away on airborne particles.

### 4.2. Chemical and Physical Characterization of Microplastics

Chemicals that are absorbed from the environment, as well as additives and polymeric raw materials formed from the plastic (such as monomers or oligomers), are the two main categories of chemicals often present in microplastics [61]. Plastics generally fall into two categories: throwaway plastic and durable plastic. Thermoplastics, such as polypropylene (PP), polycarbonate (PC), polyarylsulfone (PSU), polystyrene (PS), thermoplastic elastomers (TPE), and polyethylene terephthalate (PET), are types of polymers that are easily manipulated and reversed by altering the temperature. Thermoplastics, such as epoxy resins, vinyl ester, polyurethane (PUR), urea formaldehyde, acrylic resin, silicone, melamine resin, phenolic resins, phenolic formaldehyde, and unsaturated polyester, are a class of plastics that cannot be modified by heating. Other examples of thermoplastics include polymethyl methacrylate (PMMA), polyvinyl chloride (PVC), polypropylene (PP), polyamides (PA), and fluoropolymer.

Physical characterization determines the particle shape, size, color, general morphology, initial type, degree of corrosion, and degree of age of microplastics by eye inspection [62], dynamic research into light scattering [63], along with the analysis of laser diffraction particle size [64]. A scanning electron microscope combined with an energy-dispersive X-ray is typically used to determine the functional groups, molecular weight, structure, and degree of polymerization of the polymers in microplastics. Mixtures of heterogeneous plastic particles with a wide variety and complex compositions can be determined by various methods [65]. Infrared spectroscopy using Fourier transform [66], Raman Effect analysis [67], thermal examination [68], mass spectrometry, etc., can be successfully used in analyzing heterogeneous and complex microplastics [69].

### 4.3. Dispersion of Microplastics in the Surroundings

**Figure 2:**
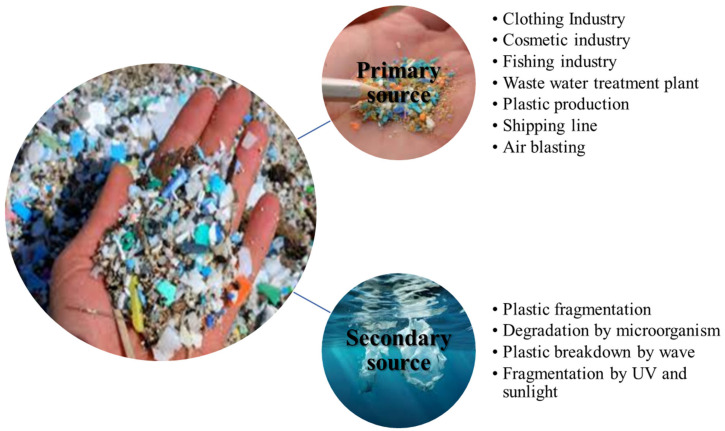
Sources of microplastics.

Depending on where they come from, microplastics in the environment might be categorized as primary or secondary. Primary microplastics are tiny particles with voluntarily added microbeads that are accidentally discharged or are byproducts of some procedure. They are purposefully made in the size range of ≤5 mm. They are utilized in the manufacturing of resin pellets and as particles for sandblasting in personal care products such as face washes, cosmetics, shampoos, and exfoliating or exfoliating toothpastes [70,71]. Usually, polyethylene is used to make them (or nylon, polypropylene, or polyethylene terephthalate). Particles known as secondary microplastics are produced when bigger plastic objects degrade and release these tiny fragments into the environment. For example, "deposits" of waste that break into smaller pieces, the shedding of fibers during washing fabrics or clothing, or the breakdown of larger plastics (like plastic bags or bottles) in nature [72]. There is a large distribution of microplastics in aquatic ecosystems, such as oceans [73], deep trenches [74,75], and sediments in offshore areas such as rivers [76], estuaries [77,78,79], lakes [80], beaches, estuaries, and islands. The aquatic environment has been a major disaster area for microplastic pollution [81]. Conventional pathways for microplastics to contaminate far-off water bodies and the deep sea include long-distance transport by water currents through rivers, wind, and ocean currents [82]. Because of the effects of tides and ocean currents, a large portion of the plastic debris discovered in the marine environment is still present along the shore. Other teams of researchers are also researching it. Numerous forms of microplastics have been shown to be present in the sediments found in lakes, estuaries, rivers, coasts, seas, and oceans. When coupled, these tiny microplastics can travel great distances to reach the ocean sink by combining them with various sediments [83]. Coastal areas may serve as an important source of microplastics that eventually find their way into the sea and ocean (Figure 2).

### 4.4. Transportation of Microplastics

Microplastics exhibit diverse transport pathways, including aquatic transport, where they are carried by ocean currents, river flows, and tides, with their buoyancy and density determining their vertical distribution in water columns. Atmospheric transport allows microplastics to travel long distances, potentially leading to their deposition in remote regions, where airborne microplastics can settle on terrestrial and aquatic ecosystems. Understanding the fate of atmospheric microplastics is often connected to those found in both land and water environments. Freshwater, marine, terrestrial, and atmospheric ecosystems are interconnected and have different sources, pathways, and sinks for microplastics. In aquatic ecosystems, microplastics are consumed by marine life and transported up the food chain, potentially impacting animals at higher trophic levels, including humans. Moreover, human-mediated transport, driven by activities such as shipping, fishing, and tourism, can facilitate the movement of microplastics between regions and ecosystems.

### 4.5. Prevalence of Microplastics in Humans and the Environment

Significant ecological problems can result from the widespread presence of microplastics in sediments and aquatic environments, which can have deleterious impacts on people and other animals [84,85,86]. Numerous heavy metals, including pollution from the marine environment, such as Al, Ag, Zn, As, Ba, Cd, Cr, Cu, Pb, Hg, Ni, Se, and Sn, are being transported via microplastics. It may also hold and absorb dangerous substances, and it can draw harmful pathogenic microorganisms, such as *Vibrio* spp., etc., from sewage.

There is evidence of microplastics in marine creatures at every stage of the food chain. The quantity of microplastic eaten differs depending on the species and the area, and it might differ greatly even within the same area. There are three basic reasons why microplastics are toxic: (1) swallowing pressures, including physical obstructions and energy expenditure during ingesting, it can deform, break, and transport microplastics, changing their size, shape, surface area, toxicity, and bioavailability, their size, shape, and surface area may alter as a result; (2) plastic additive leaks, such as plasticizers; and (3) pollution linked to microplastic [85,87,88]. It can have a variety of effects on aquatic species if consumed [89,90,91]. According to a recent study, the major ways that microplastics’ individual toxicity displays itself are through physical harm, obstruction of the gastrointestinal system, and slowed absorption rates. All of these factors have an impact on an organism’s ability to grow and develop, as well as on endocrine system abnormalities, reproductive issues, neurological ailments, and, eventually, mortality. For instance, the stomach, intestine, and/or other tissues of seals, herring, cod, whiting, and clams have been reported to contain microplastics [92]. Microplastics are known to be consumed by marine species along with their food, and there are signs that certain animals consume microplastics because they are the same size as their typical diet, such as algae. The consumption of seafood may be another way that humans are exposed to microplastics.

### 4.6. Toxic Effects

The effect on human health depends on the magnitude of the exposure concentrations. Microplastic generally affects the nervous system [93] gastrointestinal tract [94,95], excretory system [96,97], respiratory tract [98,99,100,101,102,103], internal organs [104], and also the placenta [105]. Numerous studies demonstrate the negative consequences of using plastic. Common adverse effects include gastrointestinal obstructions, which may create a feeling of excessive fullness, as well as both internal and external damage to the human body. Ingestion of micro- and nano-sized plastics is potentially associated with three types of harm: (1) physical effects associated with ingestion similar to macroplastics (but in the case of smaller organisms), (2) harmful effects brought on by the discharge of dangerous materials and also from their intended application as a raw material for the synthesis of polymers; and (3) a hazardous reaction to substances that are unintentionally adsorbed on microplastics. Rivers are significant pathways for microplastic transportation into seas and oceans [106]. Studies have documented microplastic presence in various rivers, including the Nile [107], Amazon [108], and Danube [109]. These findings highlight the need for further research on microplastic pollution in freshwater ecosystems and the development of effective mitigation strategies.

**Figure 3:**
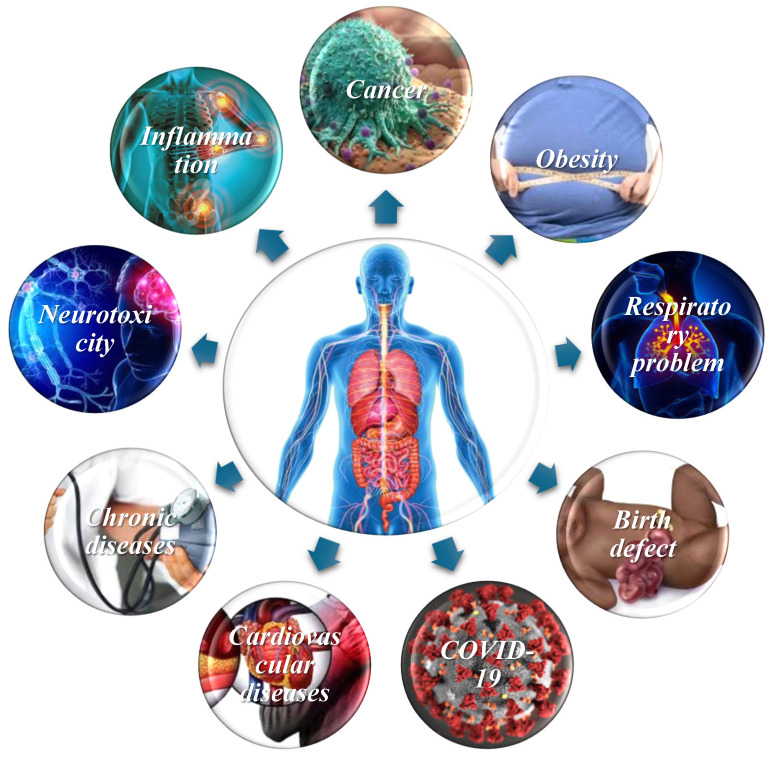
Impact of microplastics on humans.

Microplastics pose a multifaceted danger to people’s health. These minuscule particles can make their way into our bodies through tainted water and food sources, accumulating in the gastrointestinal tract and potentially causing a range of health issues. Within the gut, microplastics may trigger irritation, inflammation, and damage to the intestinal lining, resulting in digestive discomfort [110]. Furthermore, emerging research [111] indicates that microplastics can disrupt the delicate balance of the gut microbiome, influencing digestion, immune function, and overall well-being. These particles can cause the body to produce more reactive oxygen species (ROS), which can lead to oxidative stress and a variety of health issues, including cancer, obesity, cardiovascular disease, birth defects, inflammation, neurotoxicity, chronic diseases, respiratory issues, and autoimmune disorders (Figure 3). They can also cause inflammatory responses within the body, contributing to various health conditions.

Additionally, microplastics may serve as surfaces for harmful microorganisms, raising concerns about infections and exposure to pathogenic bacteria and viruses like COVID-19. Beyond the gut, there is growing evidence suggesting that microplastics may translocate to other organs and tissues, potentially impacting various organ systems. Their interaction with the immune system further complicates matters, potentially resulting in chronic immune activation or dysregulation with consequential health implications [112]. The complex interplay between microplastics and human health necessitates ongoing research to fully understand and mitigate the risks they pose (Figure 3).

## 5. Treatment and Mitigation Strategies

Advanced filtration systems, bioremediation strategies, and circular economy approaches are all various remediation approaches as solutions to microplastic pollution. Adsorption is a crucial element in many biological, chemical, and physical procedures related to the treatment of wastewater and water reclamation. It operates through many processes influenced by factors within phase boundaries. These pressures can impact and facilitate adsorption, potentially resulting in feedback loops. Adsorbents display a diverse array of surface architectures and chemical compositions, reflecting their wide array of applications. Materials such as mesoporous silica, minerals, hybrid particles, carbon nanoparticles, zeolites, activated carbons, and inorganic–organic modified bentonite are utilized for adsorption to eliminate microplastics. These adsorbents possess inherent limitations, including the inability to efficiently remove minuscule constituents from the pretreated matrix despite their high adsorption capacities. Zeolites, activated carbons, carbon nanoparticles, and inorganic–organic modified bentonite are important examples of adsorbents [113].

The substance-activated carbon (AC) is frequently used in adsorption applications due to its extensive specific surface area and minimal surface polarity, both of which augment its capacity to effectively adsorb microplastics [114].Nonetheless, notable drawbacks of AC include its chaotic structure, its incapacity to accept pores exceeding 2 nm, and its irregular distribution of pore dimensions. Adsorption is facilitated by the solution’s electrolyte content by imparting a positive surface charge to the carbon, enhancing the binding between the adsorbent and the adsorbate. Therefore, while a neutral carbon surface is optimal, a positively charged surface enhances effective adsorption. Activated carbon is commonly employed in commercial applications to enhance the quantity of groups that possess acidic oxygen and surface charge density. These modifications can be accomplished through chemical procedures such as oxidation or thermal treatment. Following heat treatment and selective modification with nitric acid, activated carbons exhibited significant improvements in microplastic adsorption. The untreated and thermally treated carbons had the highest equilibrium adsorption capacities, found at 382.12 and 432.34 mg/g, in that order. Microplastic adsorption was impacted by pH and temperature; when the temperature increased from 288 to 318 K, less microplastic was able to be adsorbed. Moreover, at pH 11.0, activated carbons showed the lowest adsorption capability [115].

Research indicates that microplastics break down rapidly in aerobic environments, implying that activated sludge might be used to biodegrade BPA. Measurements of the oxygen absorption rate of activated sludge at 22 degrees Celsius show that 10 percent of microplastics decompose in 4.7 to 5.2 days. Additional research revealed that by day 28, a maximum of 93.1% of BPA had decomposed, with a range of 77.1% to 92.3% decomposition after 10 days. Biodegradation commenced rapidly after a 3.4-day lag and continued across a wide range of influent microplastic concentrations (0.05–550,000 µg/L). In aquatic environments, Gram-negative bacteria have been shown to decompose microplastics, which they use as their sole carbon and energy source. Research investigating these bacteria’s metabolism of microplastics uncovered two main metabolites: (4,5-dimethylphenyl)-1-propanol and 2,2-bis(4,5-dimethylphenyl). The major process yielded 4-hydroxyacetophenone and 4-hydroxybenzoic acid, whereas the minor pathway yielded 1,2-propanediol. The oxidative rearrangement that results in the production of these chemicals is believed to be assisted by aerobic bacteria. Studies with Vibrio fischeri show that microplastic biodegradation also detoxifies the chemical, even though microplastic and certain BPA metabolites share estrogenic properties. What this means is that biodegradation acts as a detoxifier [116,117,118]. 

Activated sludge systems often contain two kinds of microbes—degraders and non-degraders—when they treat wastewater that contains xenobiotics, like microplastics. Although degraders may not be present initially, non-degraders could adapt to utilize different metabolic routes. Conversely, internal degradation mechanisms cause the eradication of some non-degraders. During acclimatization, there is a lag period called the “lag phase” that comes before the compound’s breakdown phase. 

The efficiency of microplastic removal in wastewater treatment is highly dependent on the biomass type utilized. In contrast to activated sludge, which is good at removing micropollutants, systems that use immobilized biomass can operate at older sludge ages and are less vulnerable to harmful chemicals. When it comes to pharmaceutical residues in wastewater, biofilms outperform activated sludge in terms of micropollutant removal speed. Biofilm stratification is caused by concentration and redox potential gradients. Most microbes in the outer layers are adept at decomposing easily biodegradable substrates, whereas those in the interior layer focus on more resistant compounds. The presence of microbial consortia that multiply slowly and are adept at decomposing refractory compounds is fostered by the longer age and higher biomass concentration in biofilms [119,120,121]. 

Not only that, activated sludge has a threshold of 5 mg/L, a tenfold increase over the level demonstrated to be harmful to immobilized biomass. Harmful substances have less of an impact on microbial development due to the reduced diffusion that happens inside biofilm layers and support holes. Biofilms have a greater toxicity threshold than activated sludge because of this characteristic, which is absent in the latter [122,123]. 

When it comes to aerobic granular wastewater treatment systems, there is a lack of data about the removal of BPA. Sequencing batch biofilter granular reactors achieved an astounding 93% BPA removal, much beyond the 72% rate observed in conventional activated sludge processes. Granules have excellent BPA removal capacity because of many factors, such as their low sludge output, high biomass concentration (40 g/L), and sludge age (up to six months). When it comes to lowering endocrine-disrupting chemicals (EDCs) like BPA, microalgal cultures provide an extra option alongside bacterial cultures. After incubating cells with a microalgal culture, one approach to BPA elimination involved causing the compound to accumulate in the cells [124].

## 6. Future Prospects of Microplastic Pollution

Concerns regarding the potential impacts of microplastic pollution on human health and physiological processes have intensified in recent years as awareness of this pressing environmental issue has escalated. Recent studies have highlighted that microplastics can be transported through the air, potentially affecting human health [125]. Previous research suggests that microplastics may affect the immune system, potentially leading to inflammation or other health issues [126]. Studies have raised alarms about how microplastics can enter the human body through various pathways, including ingestion, inhalation, and dermal contact. This growing attention underscores the urgency for research to better understand the long-term effects of microplastics on human biology and to formulate effective strategies for mitigating their presence in our environment. Recent research efforts are uncovering a deeper understanding of microplastics and their potential consequences for both the environment and human health. Researchers have also explored the use of magnetic nanoparticles to remove microplastics from water. As studies continue to explore the origins, distribution, and accumulation of microplastics, scientists are increasingly aware of their pervasive presence in ecosystems, waterways, and the food chain as discussed above. Researchers are exploring strategies to combat microplastic pollution, including promoting the creation of sustainable, circular economies to minimize plastic waste [127]. These ongoing and incoming investigations aim to comprehend not only the ecological ramifications but also the long-term health effects that microplastics may pose to living organisms, highlighting the urgent need for effective strategies to mitigate their impact. One of the emerging concerns is inhalation exposure as airborne microplastics have been identified in the atmosphere [128]. These minute particles could potentially be inhaled, settling within the respiratory tract and raising apprehensions about respiratory issues and inflammation [129]. Therefore, future studies on microplastics should delve deeper into the levels of inhalation exposure individuals experience, assessing not only the extent of this exposure but also the potential respiratory health effects associated with it [130]. Additionally, it will be essential to evaluate the biological mechanisms by which microplastics may affect respiratory health, considering factors such as toxicity, inflammation, and potential long-term impacts on various respiratory and cardiovascular organ systems. By adopting a comprehensive approach, future research can provide valuable insights and inform public health policies and environmental regulations aimed at mitigating the risks associated with microplastic pollution. Another pivotal focus area is the effects of microplastics on the presence of microorganisms in the gut [131]. The gastrointestinal system is prone to the accumulation of these particles, which may upset the delicate balance of the gut microbiota, which is crucial to human health. Changes in the makeup of the gut microbiota may have an impact on the immunological response, metabolism, and digestion, among other functions, and may be a factor in a variety of health issues. Concerns regarding chronic exposure and related health hazards are also raised by the bioaccumulation and biomagnification of microplastics through aquatic creatures, which allows them to enter the human food chain through seafood intake. Thus, forthcoming research will delve into the intricate interactions between microplastics, chemical contaminants, and human health [132].

## 7. Conclusions

Microplastics have a very high potential for bioaccumulation because of their size. Many studies show that microplastics are now a ubiquitous pollutant in the aquatic environment. It is found in beach sediments, subsea and deep sediments, both in the water column and on the surface of the water. Microplastics, like many environmental toxins, typically pass through the bodies of most organisms without causing significant accumulation. This suggests that their harmful effects are often related to the levels of exposure rather than the presence of the particles themselves. As articulated in classical toxicology, “the dose makes the poison”, emphasizing that the impact of a toxic substance depends greatly on the quantity and duration of exposure. In the context of microplastics, this principle underscores the urgent need for comprehensive research and improved methodologies to assess the exposure levels of both humans and other living organisms. Enhanced understanding of how microplastics interact within biological systems is essential for evaluating potential health risks and developing effective strategies to mitigate their impact on ecosystems and human health. Different exposure and effect pathways require additional investigation. For instance, if microplastics are ingested, they can block the gastrointestinal tract, impairing digestion and absorption processes. Additionally, they may cause physical damage to blood vessels, leading to inflammation and stress. Furthermore, microplastics can decrease energy supply and disrupt respiratory processes.

Quantifying microplastic contamination, evaluating its effects on human health and marine life, and creating cutting-edge remediation methods are the main goals of the study. Establishing sample and analysis procedures, creating consistent data formats, and defining terminology and classification schemes are all examples of standardization requirements. Global collaboration, extended producer responsibility, reducing the use of single-use plastics, and raising awareness and educating people are all examples of policy integration. We can successfully reduce microplastic pollution and safeguard human health and marine life by combining policies, standardizing procedures, and giving priority to research.

New research techniques and a range of educational initiatives are needed to manage environmental conservation and shield the ecosystem from these dangerous polymers. Raising public awareness of the detrimental consequences of microplastics is a pressing need in this industry. This would promote a number of developments aimed at lowering the consumption and usage of plastic and its derivatives. Recycling and gathering plastic pieces are the most crucial strategy to reduce the amount of plastic that enters the ecosystem. The wisest course of action is to cease production and switch to plastic items in order to prevent further risk.

## Figures and Tables

**Table 1:** Different types, sources, and uses of plastics (based on the constituents and kind of materials used in their production) [17,18].

Symbols	Types of Plastics	Properties	Sources	Common Uses
** 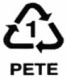 **	Polyethylene erephthalate (PET or PETE or olyester)	Wrinkle-free fiber, clear, tough, solvent resistant, barrier to gas and moisture, softens at 80 °C.	Terephthalic acid (TPA) and ethylene glycol (EG)	Soft drinks, water bottles, containers, salad dressing, biscuit trays, and salad domes.
** 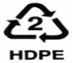 **	High-ensity olyethylene (HDPE)	Long, almost unbranched polymer chains make them really dense and, therefore, stronger and thicker than PET.	Petroleum, specifically from the ethylene monomer extracted from crude oil	Used for grocery bags, opaque milk, juice containers, shampoo bottles, and medicine bottles.
** 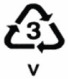 **	Polyvinyl hloride (PVC)	Strong, hard, softens at 80 °C, can be transparent, and soluble.	Salt and oil	Used in toys, blister packs, wrappers, detergent bottles, loose-leaf binders, blood bags, and medical tubing.
** 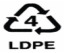 **	Low-ensity olyethylene (LDPE)	Soft, flexible, waxy surface, transparent, softens at 70 °C, easy to scratch.	Monomer ethylene, derived from petroleum or natural gas	Used for bags (grocery, dry cleaning, bread, freezer bags, newspapers, trash), plastic wrap; lids for paper milk cartons and hot and cold drinks; some squeeze bottles (honey, mustard), food containers, and container lids. Also used to cover wires and cables.
** 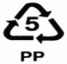 **	Polypropylene (PP)	Hard and semi-transparent, softens at 140 °C, semi-transparent, resistant to solvents, versatile.	Petroleum	Used for hot food containers.
** 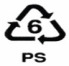 **	Polystyrene (PS)	Transparent, glassy, rigid, opaque, semi-hard, softens at 95 °C, affected by fats, acids, and solvents, but resistant to alkalis and salt solutions, low water absorption, if not pigmented, clear, odorless, and tasteless.	Non-renewable fossil fuels and synthetic chemicals	Used for food containers, egg cartons, disposable cups, bowls, packaging, and bicycle helmets.
** 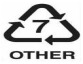 **	Other	Other plastics that have all the properties of resins and several materials (e.g., laminates) depending on the plastic or combination of plastics types.	Edible fish, drinking water, salt, honey, air, mussels, oysters, sea mullet, canned sardines, and sparts	Components for cars and home appliances, computers, electronics, cold bottles, and packaging.

**Table 2:** Summary of the included studies and database results.

Medical Search Engines	Results
#Pubed database	210
# Medline	85
#NDSL (National Digital Science Library)	34
Total database search	329
Document eliminated	246
Included study	32

**Table 3:** Result outcome of selected studies [19,20,21,22,23,24,25,26,27,28,29,30,31,32,33,34,35,36,37,38,39,40,41,42,43,44,45,46,47,48,49,50].

SL. No.	Author Details	Year	Purpose of Study	The Results of the Study
1	Tiwari B. R. et al. [19]	2023	Medical applications of plastic, such as PPE and packaging materials, have increased dramatically as a result of the COVID-19 epidemic.	Health problems like cancer, diabetes, and allergic reactions can result from the buildup of microplastics in the human body; therefore, efficient detection and disposal techniques are required.
2	Sun M. Y. et al. [20]	2024	A prospective view for MP research is provided by recent studies that examine the sources, distribution, exposure pathways, harmful effects, and processes of MPs on humans, animals, and plants.	Microplastics are produced and managed in garbage, which has a negative impact on the environment and living things. MPs have the ability to absorb and consume contaminants, which can impact metabolism and health.
3	Wang T. et al. [21]	2023	The study looks at microplastic pollution in different Tibetan Plateau habitats, evaluating its source, fate, and possible ecological impacts in addition to its spatial distribution.	Even at Mount Everest, microplastics are present in greater quantities than in the ocean system in the biotic and abiotic elements of the Tibetan Plateau.
4	Lee M. et al. [22]	2022	In order to evaluate the spread of microplastic contamination across the different environmental pillars—aqueous, terrestrial, airborne, bio-organism, and human—the article carried out a comprehensive literature analysis.	Faster rates of plastic breakdown have been found in recent studies, exposing microplastics as a new source of harmful contamination that is spreading from the ocean to the soil, groundwater, air, and food chain.
5	Goyal T. et al. [23]	2023	Current information on possible sources of MP in soil, water, and air, as well as their analysis techniques, effects on human health, and remediation techniques, is included in this review.	MPs are present in a variety of sources, including drinking water, ocean, beach sand, agricultural soils, wastewater treatment plant effluent, and the atmosphere. They are also becoming more prevalent in food products and soil ecosystems.
6	Vivekananda A. C. et al. [24]	2021	The origins, movement, impacts, worldwide regulatory frameworks, methods of detection, and management approaches of microplastics in aquatic environments.	Microplastics are a major ecological and health hazard since they can enter aquatic life and the food chain and are released from commonplace objects, industry, and wastewater treatment facilities.
7	Li Y. et al. [25]	2023	According to the literature review, microplastics are commonly detected in human and environmental samples.	Oxidative stress, DNA damage, organ malfunction, metabolic abnormalities, immunological response, neurotoxicity, reproductive and developmental toxicity, and possible chronic diseases are among the harmful impacts of microplastics that draw attention to study gaps and future development objectives.
8	Goswami S. et al. [26]	2024	In-depth analysis of microplastics is given in this review, which looks at their chemistry, origins, exposure to humans, toxicity, and molecular potential for cancer.	Environmental pollutants mimic endocrine mediators like estrogen and androgen and affect cell-cycle proteins, redox homeostasis, gene expression, and the state of inflammatory mediators, all of which contribute to carcinogenesis.
9	Luo D. et al. [27]	2024	Recent studies on the sampling and detection, origin and properties, transport, and destiny of atmospheric MNPs are compiled in this review.	According to studies, inhaling MNP might result in oxidative stress, apoptosis, and negative immunological responses, which may cause cardiovascular disorders as well as anomalies in reproduction and development.
10	Sharma S., Chatterjee S. [28]	2017	Microplastics build up in the cells and tissues of marine creatures, causing long-term biological impacts.	Human health and marine biota are at risk from microplastics, which is why excessive plastic use must be controlled, and laws and regulations governing litter sources must be put in place.
11	Lin Y. D. et al. [29]	2023	This review explores the complexities of microplastics, including their sources, absorption, and harmful effects on people and the environment.	The environment has been severely harmed by the overproduction, use, and disposal of plastic products, which is why governments around the world are taking immediate action to reduce plastic employment.
12	Haldar S. et al. [30]	2023	The review examines the genesis, bioaccumulation, and toxicity of nanoplastics on human and environmental systems, and it makes recommendations for practical ways to reduce plastic pollution.	The primary ways that NPs are exposed are by eating and inhalation; absorption mechanisms and cytotoxic consequences are explored. There isnot much research on NP toxicity issues, though.
13	Hoang W. et al. [31]	2024	Investigating the remarkable resilience and resilience to biodegradation of microplastics in the environment is the goal of the project.	Small and easily consumed by soil organisms, microplastic trash can harm plants and hinder their growth; thus, efforts must be made to lessen these effects for the sake of environmental health and agricultural sustainability.
14	Sridharan S.et al. [32]	2021	The assessment identifies data limitations and knowledge gaps regarding the impact of atmospheric transport and particle plastics on urban air quality, assessing whether contamination is trivial or significant.	Though their quantity and importance to human health are yet unknown, airborne particulate matter (PM) has a major impact on urban air quality and atmospheric transmission to pristine environments.
15	Huang W. et al. [33]	2021	This review highlights scientific advancements on the trophic transfer of pollutants and microplastics along the aquatic food chain while analyzing the effects of microplastics on aquatic species at different trophic levels.	With the goal of improving our knowledge of microplastics’ impacts on aquatic ecology, this study investigates the possible health concerns associated with them, specifically through exposure to food chains and diet.
16	Naik R. K. et al. [34]	2019	This paper looks at how microplastics can spread harmful chemicals, metals, antibiotics, pathogenic bacteria, and dinoflagellates that produce HABs from one continent to another through ballast water.	Due to a surge in bacterial illness outbreaks and HABs, microplastics in ballast waters may pose a major health risk by aiding in the development and spread of drug-resistant human infections.
17	Khalid N. et al. [35]	2023	This analysis addresses current knowledge gaps and suggests future directions for farmland soil research by examining the application of plastic mulch films in agroecosystems.	MPs have the ability to impact human health as they go up and down the food chain’s trophic levels.
18	Lin X. H. et al. [36]	2020	This study covers the idea, classification, source, impact on human health, and proposed remedies to China’s microplastic pollution, with the goal of guiding risk assessment and regulation design.	Microplastics, a growing environmental pollutant, are hazardous to human health and the environment due to their widespread use, manufacture, and degradation.
19	Ye J. et al. [37]	2024	Nanoplastics’ origins, dispersion, human exposure, and accumulation in reproductive systems, with a focus on their consequences on animal and human reproductive health.	Research indicates that nanoplastics have detrimental impacts on human and aquatic reproductive systems, but little is known about how they affect mammals and people.
20	Masud R. I. et al. [38]	2023	The sources, amount, and destiny of biomedical waste are reviewed in this article, with particular attention to the recent increase in MPs and their detrimental impacts on both aquatic and terrestrial life.	Chronic diseases are brought on by MP intake in humans; thus, it is imperative that biomedical waste be urgently controlled and managed to lower MP pollution and lower threats to human health and the environment.
21	Zhu Y. et al. [39]	2024	The risks and toxicity mechanisms of MPs/NPs in nine biological systems are systematically understood thanks to this review.	Ingestion and inhalation of microplastics can have detrimental effects on the respiratory and digestive systems, resulting in inflammation, oxidative stress, and changes in metabolism.
22	V. G. et al. [40]	2023	The composition and interactions of MPs in landfill leachate, possible mitigating strategies, and the difficulties of present leachate treatment for MP elimination are all covered in this paper.	Because landfill leachate contains harmful pollutants and antibiotic resistance genes, which are now known to be emerging contaminants, MPs can pose health problems.
23	Thushari G. G. N., Senevirathna J. D. M. [41]	2020	The goal of this review paper is to investigate several facets of plastic contamination in marine and coastal settings.	In order to increase awareness of a plastic-free, healthy blue ocean in the near future, this article focuses on the existing level of plastic pollution in the marine ecosystem.
24	Mitrano D. M. et al. [42]	2021	This perspective investigates nanoplastics’ involvement in global plastic pollution, including their sources, hazards, and potential similarities to other nanosized environmental items such as manmade nanomaterials and natural colloids.	The physical and chemical properties of various plastic pollution sizes (macroplastics, microplastics, and nanoplastics) will result in distinct fates and hazards.
25	Wang Y. et al. [43]	2021	This study gives a thorough summary of existing information and makes recommendations for future research to improve our understanding of airborne microplastics and their potential human health hazards.	Airborne microplastics, mostly derived from synthetic fabrics and fibers, contribute significantly to pollution in aquatic and soil habitats, posing a health concern to humans.
26	Rahaman M. N. et al. [44]	2023	This article summarizes the global distribution and source of MP inside coral reefs.	Investigations should properly understand the distribution, destiny, and impacts of MPs on human and coral health, as well as their environmental risks.
27	Lee W. K., Thévenod F. [45]	2020	This review critically investigates cadmium’s effects on organellar structure and function, with an emphasis on the disruption of organelle physiology in vertebrates.	Interorganellar communication is essential for proper cell function and includes signaling molecules, organelle exchange, mechanical force, shape changes, and membrane contact sites.
28	Zhang Q. et al. [46]	2020	46 articles on the quantity, sources, and analytical techniques of microplastics in air, drinking water, and table salt as well as their possible paths of accumulation and translocation within the human body were examined in this study.	An estimated 0–7.3×104 pieces of microplastics are consumed annually per person, posing serious risks to the digestive and respiratory systems, especially when inhaled indoors.
29	Cavazzoli S. et al. [47]	2023	This paper evaluates and criticizes popular techniques for examining MNPs in environmental, sludge, and sewage samples, and it makes recommendations for possible fixes to any issues found.	Human health and the sustainability of the ecosystem are at danger due to the toxicological concerns posed by MNPs and their harmful pollutants. It is vital for consumers to understand whatever happens to them in sewage treatment facilities.
30	Zhou Y. et al. [48]	2020	In order to quantify soil microplastics (MPs) and comprehend how MPs, properties, and environmental factors interact, a standardized approach for MP analysis is suggested.	The evaluation helps establish waste management and remediation strategies for regional soil MP pollution by functioning as a roadmap for soil MP research techniques and frameworks.
31	Li T. et al. [49]	2024	The life cycle, survival, mutations, loads, shedding, transmission, infection, re-assortment, interference, abundance, and host vulnerability of viruses are all assessed in this review in relation to chemical and biological contaminants.	Understanding how environmental pollutants affect animal viruses could help further research on the harmony of viruses, animals, humans, and ecosystems to maintain human health.
32	Pham V. H. T. et al. [50]	2024	The study emphasizes the value of employing extremophiles to accelerate the breakdown of plastic, paying particular attention to elements like amyloid precursor protein as well as cell hydrophobicity.	The goal of this review is to improve the release of carbon dioxide and future H2/CH4 synthesis by bioprospecting for unknown methods for plastic degradation bioproducts.

## Data Availability

Data sharing is not relevant to this paper because it is a review article. This publication does not contain any newly produced or analyzed data.

## Abbreviations

The following abbreviations are used in this manuscript:

ROS	Reactive Oxygen Species
NDSL	National Digital Science Library
PRISMA	Preferred Reporting Items for Systematic Reviews and Meta-Analysis
PP	Polypropylene
PC	Polycarbonate
PSU	Polyarylsulfone
PS	Polystyrene
TPE	Thermoplastic elastomers
PET	Polyethylene terephthalate
PUR	Polyurethane
PMMA	Polymethyl methacrylate
PVC	Polyvinyl chloride
PP	Polypropylene
PA	Polyamides
AC	Activated carbon
ECs	Emerging contaminants
EDCs	Endocrine-disrupting chemicals
